# Photocathodic Protection of 304 Stainless Steel by Bi_2_S_3_/TiO_2_ Nanotube Films Under Visible Light

**DOI:** 10.1186/s11671-017-1863-9

**Published:** 2017-01-31

**Authors:** Hong Li, Xiutong Wang, Qinyi Wei, Baorong Hou

**Affiliations:** 1 Key Laboratory of Marine Environmental Corrosion and Bio-fouling, Institute of Oceanology, Chinese Academy of Sciences, No. 7, Nanhai Road, Qingdao, 266071 China; 2 University of Chinese Academy of Sciences, Beijing, 100049 China

**Keywords:** TiO_2_ nanotube, Bi_2_S_3_, Stainless steel, Photocathodic protection

## Abstract

We report the preparation of TiO_2_ nanotubes coupled with a narrow bandgap semiconductor, i.e., Bi_2_S_3_, to improve the photocathodic protection property of TiO_2_ for metals under visible light. Bi_2_S_3_/TiO_2_ nanotube films were successfully synthesized using the successive ionic layer adsorption and reaction (SILAR) method. The morphology and structure of the composite films were studied by scanning electron microscopy and X-ray diffraction, respectively. UV–visible diffuse reflectance spectra were recorded to analyze the optical absorption property of the composite films. In addition, the influence of Bi_2_S_3_ deposition cycles on the photoelectrochemical and photocathodic protection properties of the composite films was also studied. Results revealed that the heterostructure comprised crystalline anatase TiO_2_ and orthorhombic Bi_2_S_3_ and exhibited a high visible light response. The photocurrent density of Bi_2_S_3_/TiO_2_ was significantly higher than that of pure TiO_2_ under visible light. The sensitization of Bi_2_S_3_ enhanced the separation efficiency of the photogenerated charges and photocathodic protection properties of TiO_2_. The Bi_2_S_3_/TiO_2_ nanotubes prepared by SILAR deposition with 20 cycles exhibited the optimal photogenerated cathodic protection performance on the 304 stainless steel under visible light.

## Background

304 stainless steel (304SS) is widely used in various industries for its good corrosion resistance and fabricability. However, this material easily deteriorates from pitting corrosion in seawater and chloride-containing solutions [1, 2]. Recently, photocathodic protection for metals has received growing attention from scientists worldwide as a promising and green technology [3–7]. TiO_2_ has been extensively investigated as a photoanode for the cathodic protection because of its high chemical stability, low cost, and nontoxicity [8–11]. However, its wide bandgap (~3.2 eV for anatase) restricts its application because of its exclusive activity only under UV irradiation (3–5% of the solar spectrum) [12, 13]. The recombination of photogenerated electrons and holes in the dark results in a low photo-quantum efficiency of TiO_2_. To overcome these defects, TiO_2_ nanotube arrays with large specific surface areas were synthesized [14–16] and various strategies were developed to expand its absorption to the visible light range. These strategies include coupling with narrow-bandgap semiconductors (ZnSe, WO_3_, SnO_2_, CdS, and Ag_2_S) [17–21], metals (Ag, Au, Cu, and Bi) [22–24], and nonmetals (N, F, and graphene) [25–27]. Bi_2_S_3_ is an attractive material because of its narrow bandgap (*E*_g_ = 1.3 eV) and high photo-to-electron conversion efficiency [28]. The Bi_2_S_3_/TiO_2_ heterostructure can reduce the recombination of photogenerated electrons and holes, and this effect would benefit the photoelectric performance of materials [29–32]. However, no research has been reported on the photogenerated cathodic protection property of Bi_2_S_3_/TiO_2_ nanotubes. Successive ionic layer adsorption and reaction (SILAR) is a promising technique with low cost and simple equipment, which can synthesize continuous and compact film at room temperature [33]. In this study, Bi_2_S_3_/TiO_2_ nanotube films served as photoanode for preventing 304SS corrosion. In the fabrication of the films, Bi_2_S_3_ nanoparticles were prepared by the SILAR method. The morphology, structure, and optical absorption property were studied by scanning electron microscopy (SEM), X-ray diffraction (XRD), and UV–visible (UV–vis) diffuse reflectance spectra, respectively. The influence of Bi_2_S_3_ deposition cycles on the photoelectrochemical and photocathodic protection properties of the composite films was also investigated in our work.

## Methods

TiO_2_ nanotubes were first fabricated by anodizing Ti foil in ethylene glycol electrolyte comprised of 0.5 wt% NH_4_F and 6 vol% H_2_O for 1.5 h and annealing at 450 °C for 1.5 h in air. Then, Bi_2_S_3_/TiO_2_ nanocomposites were prepared through the alternate immersion of TiO_2_/Ti substrate in the anionic and cationic precursor solutions at room temperature. The cationic precursor solution was composed of 0.01 M Bi(NO_3_)_3_ dissolved in 50 ml of acetone. Meanwhile, the anionic precursor solution was composed of 0.01 M Na_2_S dissolved in 50 ml of methanol. The TiO_2_/Ti substrate was first dipped into the cationic precursor solution for 20 s, and then dipped into the anionic precursor solution for 20 s, rinsed, and dried in air. The Bi_2_S_3_ synthesized in 10, 20, and 30 deposition cycles were assigned as BST-10, BST-20, and BST-30, respectively.

**Fig. 1:**
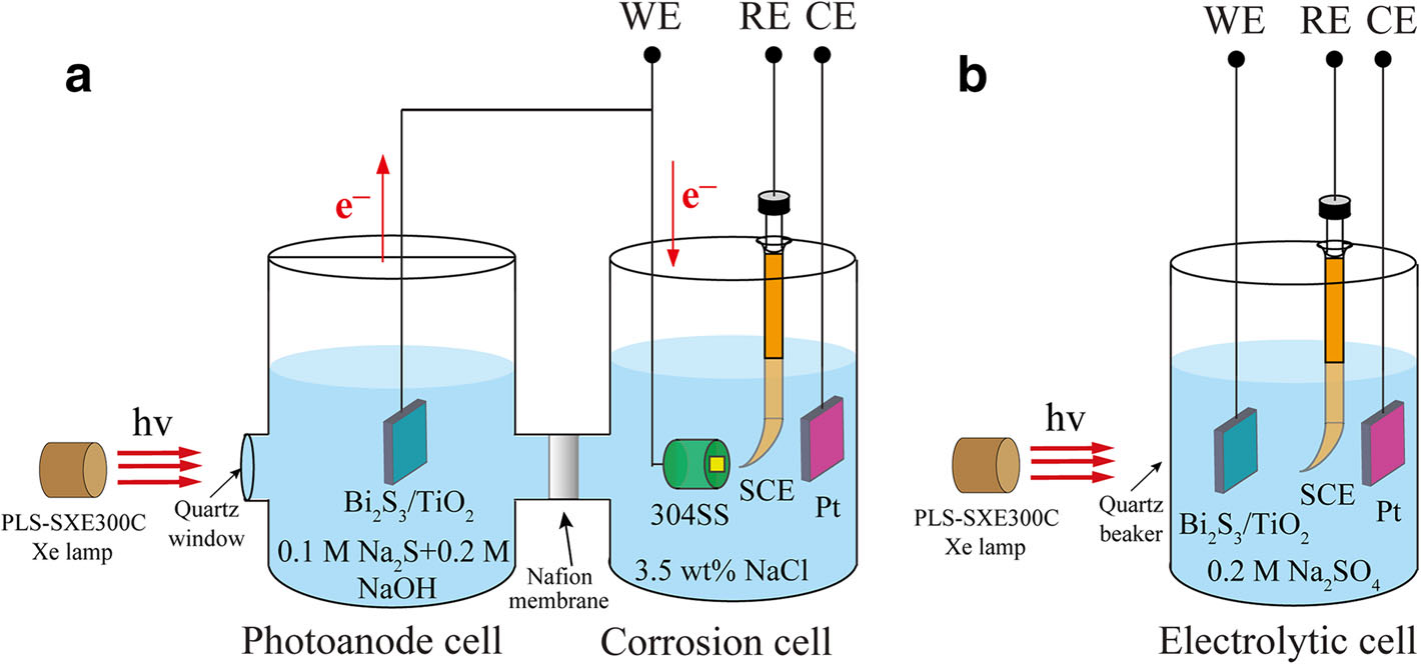
Schematic sketches of experimental devices for photoelectrochemical characterization of OCPs (**a**) and transient photocurrent curves (**b**)

The morphology of the samples was investigated by SEM (Hitachi S-4800, Japan). The structure of the samples was examined by XRD (Bruker AXSD8 Advance, Germany). The UV–vis diffuse reflectance spectra were obtained on an UV–vis diffuse reflectance spectrophotometer (Hitachi UH4150, Japan). Photoelectrochemical experiments were conducted using a potentiostat/galvanostat (PARSTAT 2273, USA) at room temperature with a Xe lamp (PLS-SXE300C, China) as the visible light source. The open-circuit potential (OCP) of different coupled photoelectrodes were investigated in a double-cell system (Fig. 1a). A TiO_2_ or Bi_2_S_3_/TiO_2_ nanotube photoelectrode was placed in a photoanode cell containing a mixed 0.1 M Na_2_S and 0.2 M NaOH solution, whereas 304SS was placed in a corrosion cell containing 3.5 wt% NaCl solution. The Pt foil, saturated calomel electrode (SCE), and coupled electrode of TiO_2_ and 304SS electrode served as the counter electrode (CE), reference electrode (RE), and working electrode (WE), respectively. Photocurrent curves were measured in 0.2 M Na_2_SO_4_ solution using an electrochemical workstation (CHI 1010C, China). The TiO_2_ or Bi_2_S_3_/TiO_2_ composite photoelectrode, SCE, and Pt foil served as the WE, RE, and CE, respectively (Fig. 1b).

## Results and Discussion

**Fig. 2:**
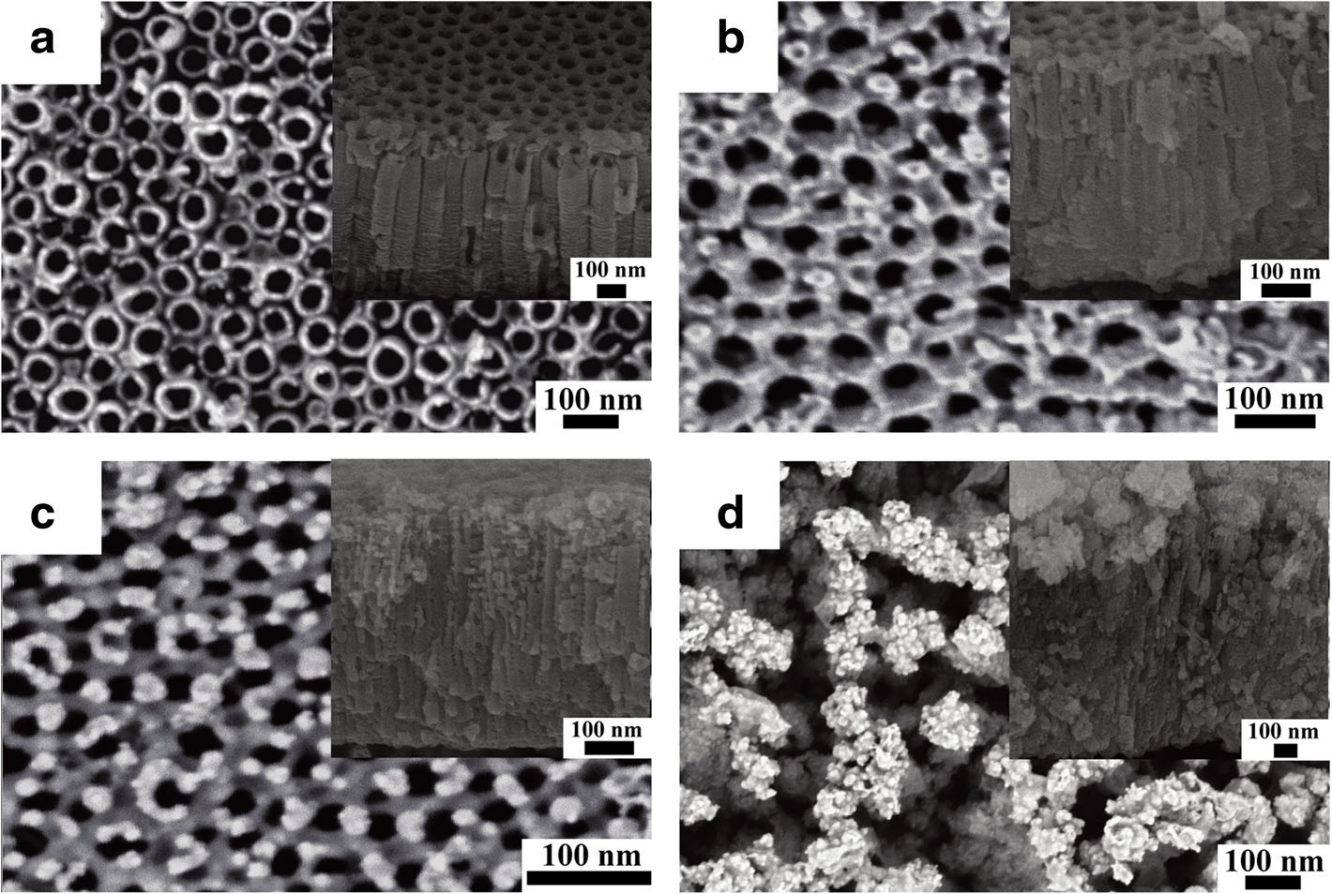
SEM images of **a** pure TiO_2_, **b** BST-10, **c** BST-20, and **d** BST-30

The morphologies of Bi_2_S_3_/TiO_2_ heterostructure were observed by SEM (Fig. 2). TiO_2_ nanotube arrays exhibited a well-ordered, high-density, and uniform tubular structure with an average diameter of 60 nm (Fig. 2a). The Bi_2_S_3_ nanoparticles were successfully deposited on TiO_2_ nanotube surfaces through the SILAR method (Figs. 2b–d). For BST-10, the particles distributed irregularly on the mouth of TiO_2_ nanotubes (Fig. 2b). When the number of Bi_2_S_3_ deposition cycle increased to 20, the Bi_2_S_3_ nanoparticles were deposited regularly on the mouth or wall of TiO_2_ nanotubes with about 15 nm in diameter. After undergoing 30 cycles, the amount of nanoparticles significantly increased, and the formation of agglomeration caused the particles to block the nanotubes.

**Fig. 3:**
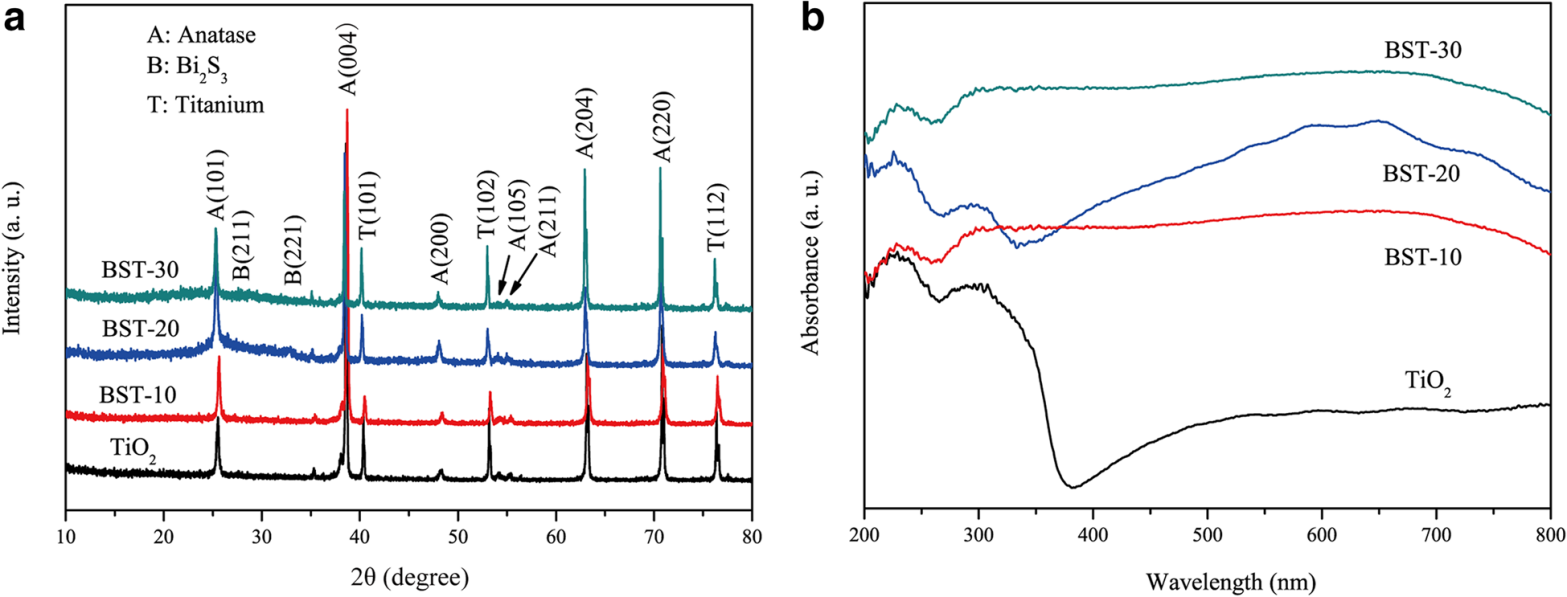
XRD patterns (**a**) and UV–vis diffuse reflectance spectra (**b**) of pure TiO_2_ and Bi_2_S_3_/TiO_2_

Figure 3a depicts the XRD patterns of TiO_2_ and Bi_2_S_3_/TiO_2_. Aside from the diffraction peaks of titanium substrate, the peaks at 25.38°, 38.03°, 48.01°, 54.05°, 55.17°, 62.71°, and 70.44° can be indexed to lattice planes (101), (004), (200), (105), (211), (204), and (220) of anatase TiO_2_, respectively (JCPDS 21-1272). Besides the TiO_2_ peaks, the peaks at 27.74° and 32.54° were attributed to lattice planes (211) and (221) of the orthorhombic Bi_2_S_3_ (JCPDS 17-0320). For Bi_2_S_3_/TiO_2_ nanocomposites, the increase in diffraction peak intensity of Bi_2_S_3_ with the deposition cycles revealed an increased amount of Bi_2_S_3_ nanoparticles on the TiO_2_ nanotubes. This finding is consistent with the SEM results.

The light absorption abilities of the synthesized Bi_2_S_3_/TiO_2_ nanotube films were assessed by UV–vis spectroscopy (Fig. 3b). Figure 3b shows that TiO_2_ nanotubes absorbed mainly in the UV range with a wavelength of about 380 nm because of the bandgap energy of anatase (3.2 eV). The spectra of Bi_2_S_3_/TiO_2_ exhibit a relatively broad and strong absorption in the visible region, indicating that the Bi_2_S_3_/TiO_2_ nanocomposite is capable of harvesting visible light and acts as a photoanode under visible light [34].

**Fig. 4:**
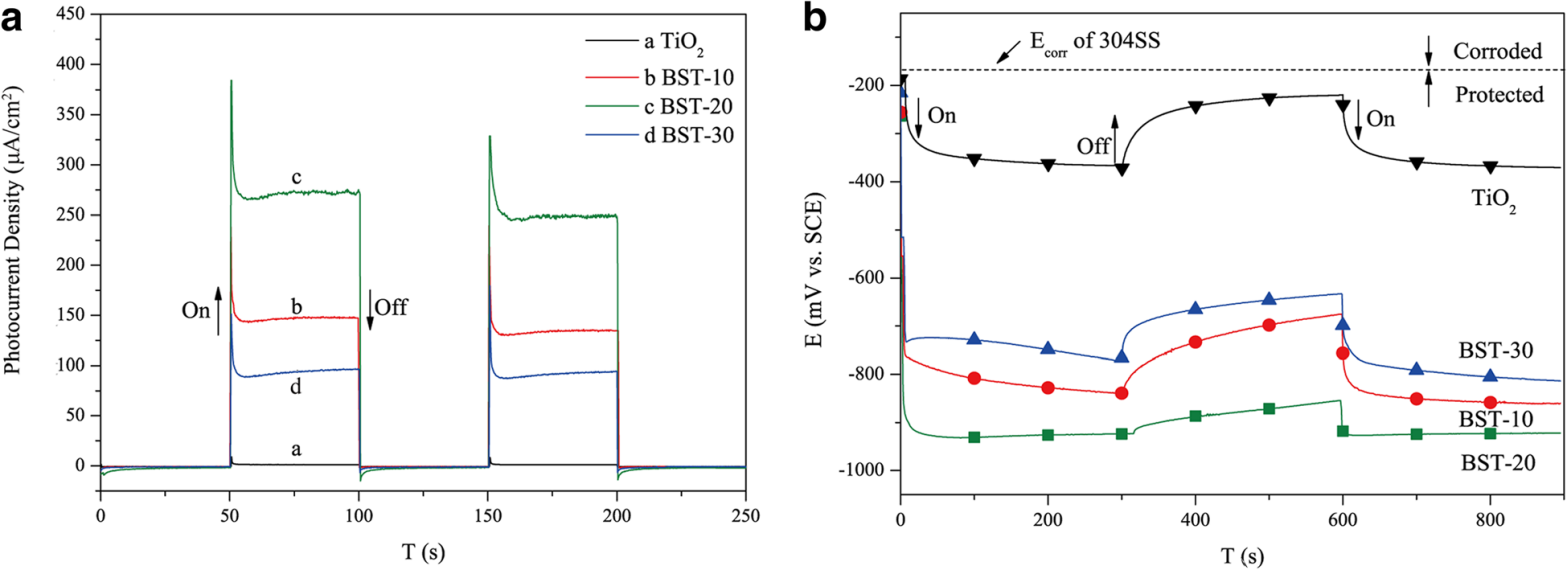
Photoresponse spectra (**a**) and OCP variations (**b**) of pure TiO_2_ and Bi_2_S_3_/TiO_2_ under intermittent irradiation

Figure 4a displays the transient photocurrent curves for TiO_2_ and Bi_2_S_3_/TiO_2_ photoelectrodes under visible light irradiation. The pure TiO_2_ nanotube photoelectrode shows nearly 0 μA/cm^2^ because of weak visible light absorption. However, after Bi_2_S_3_ nanoparticle sensitization, the transient photocurrent densities of Bi_2_S_3_/TiO_2_ exhibited an obvious increase, implying that the Bi_2_S_3_/TiO_2_ nanocomposite is capable of utilizing visible light and the heterostructure promotes the separation of photogenerated electrons and holes [35]. The transient photocurrent density of BST-20 (249 μA/cm^2^) was higher than that of BST-10 (134 μA/cm^2^) and BST-30 (92 μA/cm^2^), indicating that BST-20 possesses an optimal separation efficiency of the photogenerated electrons and holes.

Figure 4b compares the photogenerated OCPs of 304SS coupled with different TiO_2_ nanotubes. When the light was on, the potentials of coupled electrodes all shifted negatively within a few seconds. This effect may be attributed to the cathodic polarization of 304SS which results from the excited photoelectrons transfer from TiO_2_ nanotubes to 304SS [36, 37]. After the light was off, the OCP of 304SS coupled to pure TiO_2_ returned to a value near the free corrosion potential of bare 304SS, indicating the invalid recombination of the photogenerated electrons and holes in the TiO_2_ [38]. By contrast, the OCPs of 304SS coupled with Bi_2_S_3_/TiO_2_ exhibited a slightly positive shift and stayed far below than the free corrosion potential of bare 304SS. The charges stored in the Bi_2_S_3_/TiO_2_ composite were released and again transferred to 304SS in the dark. The negative shift of potentials is reportedly an important parameter for evaluating the separation efficiency of photogenerated charges [39, 40]. The increased negative shift of the potentials indicates the increased effectiveness of the cathodic protection of photoanodes. Under visible light, the shift of potentials can be ranked in the following order: TiO_2_ (150 mV vs. SCE) < BST-30 (534 mV vs. SCE) < BST-10 (572 mV vs. SCE) < BST-20 (662 mV vs. SCE). Hence, BST-20 possesses the optimal photocathodic protection property for 304SS. This result may be due to the fact that the active sites and light harvesting increased with the rising Bi_2_S_3_ amount. However, the excessive Bi_2_S_3_ particles served as the recombination sites of the electrons and holes, which hindered the charge transfer from the Bi_2_S_3_/TiO_2_ composite to 304SS.

**Fig. 5:**
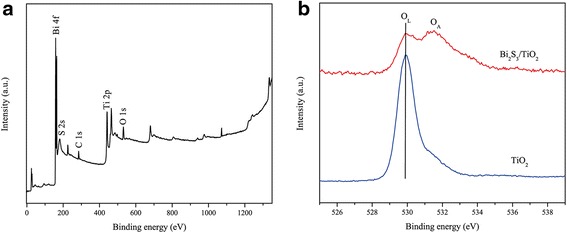
XPS survey spectra of the synthesized Bi_2_S_3_/TiO_2_ (**a**) and high-resolution XPS spectra of O 1s of TiO_2_ and Bi_2_S_3_/TiO_2_ (**b**)

The X-ray photoelectron spectroscopy (XPS) was measured to investigate the chemical compositions and states of Bi_2_S_3_/TiO_2_ (BST-20). The XPS survey spectra revealed the existence of Bi, S, Ti, and O components, in addition to C contaminants (Fig. 5a). As shown in Fig. 5b, the XPS peaks of O 1s at 529.7 eV were analyzed from the lattice oxygen (O_L_) in TiO_2_. The peak at 531.6 eV was derived from the adsorbed oxygen (O_A_). The O_A_ was composed of OH species or weak bonding oxygen on the composite surface. The presence of O_A_ was ascribed to the generation of oxygen vacancy on the sample surface. This suggests that the Bi_2_S_3_/TiO_2_ composite exhibits higher photocathodic protection properties than TiO_2_.

**Fig. 6:**
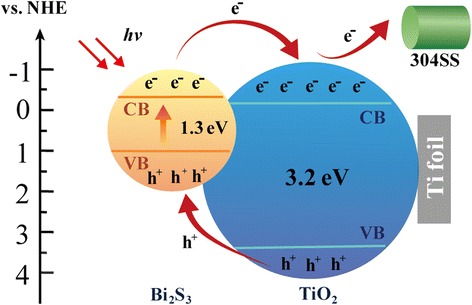
Schematic representation of the electron transfer processes in Bi_2_S_3_/TiO_2_

Figure 6 shows the schematic diagram of the photoelectric conversion and transportation processes in the Bi_2_S_3_/TiO_2_ composite. The Bi_2_S_3_ nanoparticles can easily absorb the photons in the visible light due to the presence of O_A_ and the suitable bandgap width of Bi_2_S_3_. When the photons were absorbed by the Bi_2_S_3_ nanoparticles, the photoexcited electrons were generated and transferred from the conduction band (CB) of Bi_2_S_3_ to the CB of TiO_2_. The photogenerated holes were then shifted from the valence band (VB) of TiO_2_ to the VB of Bi_2_S_3_. When Na_2_S served as a hole-trapping agent, the photogenerated charges were effectively separated. The electrons were finally transferred to the 304SS electrode, and the potential of the 304SS electrode negatively shifted. The 304SS was prevented from corrosion by Bi_2_S_3_/TiO_2_ under visible light. Therefore, the more efficient separation of the photogenerated charges in the composite would accelerate the oxidation and reduction reactions and, hence, generate a higher photocathodic protection activity than TiO_2_.

## Conclusions

In summary, Bi_2_S_3_-nanoparticle-decorated TiO_2_ nanotubes were successfully synthesized through the electrochemical anodization and SILAR method. The sensitization of Bi_2_S_3_ significantly extended the spectral response from UV to the visible region. Consequently, the composite showed higher photocurrents and cathodic protection performance than pure TiO_2_. With increased number of Bi_2_S_3_ deposition cycles, the increasing grain size and loading of the Bi_2_S_3_ nanoparticles significantly affected the photocathodic protection activity of the Bi_2_S_3_/TiO_2_ nanocomposite. The Bi_2_S_3_/TiO_2_ nanotubes prepared by SILAR deposition with 20 cycles exhibited the optimal photocathodic protection property.

## Abbreviations

304SS: 304 stainless steel
CB: Conduction band
CE: Counter electrode
O_A_: Adsorbed oxygen
OCP: Open-circuit potential
O_L_: Lattice oxygen
RE: Reference electrode
SCE: Saturated calomel electrode
SEM: Scanning electron microscopy
SILAR: Successive ionic layer adsorption and reaction
VB: Valence band
WE: Working electrode
XRD: X-ray diffraction
